# Reports of Societies

**Published:** 1875-01

**Authors:** 


### Reports of Societies.

### CHICAGO SOCIETY OF PHYSICIANS AND SURGEONS.

*Regular Meeting, Nov.* 23, 1874.

(Reported by RALPH E. STARKWEATHER, M.D.)

Dr. John Bartlett, President, in the chair.

Prof. De Lafontaine gave to the Society his views upon the hydrate of chloral and its action, together with details of experiments made by him, and by Dr. C. P. Simon, upon rabbits, cats and sheep, by the hypodermic injection of the hydrate of chloral.

Prof. De Lafontaine thought that the hydrate of chloral diminished the available oxygen of the blood by the poisoning of the red discs by the carbonic oxide gas set free in the action of chloral, that a person frequently taking chloral suffers from its effects as he would from frequent venesection. In poisoning by chloral, the blood taken from the jugular vein, before death, is red as carmine; after death, for some time, it will not coagulate, and the condition is like that of one who dies from suffocation.

The test, by the spectroscope, for the carbonic oxide, was then given. If the blood is healthy, there will be the two dark bands of absorption, yellow and green: if the blood has been poisoned by the carbonic oxide, the bands will likewise be the same; but when treated by the hydro-sulphide of ammonium, the two dark bands coalesce in the normal blood; but there is no such effect whatever in that which has been poisoned. Chloral changes the form of the red corpuscles; their shape is lost, and they become elongated, shriveled and rugose. Frequent use of chloral will ultimately ruin the health, as much so as the constant use of morphine. Dr. Simon, in relating the experiments he had made upon sheep, remarked that he was not quite of the same opinion as to the danger of using hydrate of chloral, as expressed by his friend, Prof. De Lafontaine. The subject of anæsthesia and chloral, and the experiments made by Dr. Simon and Prof. De Lafontaine, were then discussed by the Society.

Dr. Owens reported a case, which he had previously reported as recovered, in which the patient has since died. It was an operation for the removal of a colloid multilocular ovarian tumor. The patient died from septicæmia. A chart of the pulse, respiration and temperature of the patient accompanied the paper; and also a report by Dr. Danforth on the microscopical examination of the tumor and its contents. We give one only of the comments on this case, made by Dr. Owens, as the article is soon to be published: "In this case, the most practiced touch could not distinguish between a colloid growth and an ovarian encysted tumor. It would be well to tap before operating, and examine the material thus brought away."

Dr. Hollister read an elaborate article upon the subject of catalepsy, illustrating the same by details of a case of rare interest, under his care at the Mercy Hospital. By request, no notes of the paper were taken, as it is expected that it will be printed in one of the medical periodicals, in its full length.

---

### CHICAGO SOCIETY OF PHYSICIANS AND SURGEONS.

*Regular Meeting, Dec.* 14, 1874.

(Reported by RALPH E. STARKWEATHER, M.D.)

Dr. John Bartlett, President, in the chair.

Dr. P. S. Hayes read a report of two cases of amenorrhœa which had been treated in the Woman's Hospital of the State of Illinois.

The first case was one of amenorrhœa from infantile uterus and ovaries, treated by dilatation, local applications and electricity.

*History.* Mrs. H., colored, twenty-three years of age, came under observation June 7, 1873. Has always been delicate, and suffered from numerous indispositions. Her first menstrual period occurred when she was seventeen years of age. Since that time at irregular periods there has been a slight sanguineous discharge from the vagina. Each month she has a return of pain in the back, limbs and head, accompanied occasionally by epistaxis. She is habitually constipated.

An examination made by Dr. A. Reeves Jackson disclosed an infantile uterus; the sound passing into the uterus measured only three-fourths of an inch; neither ovary was distinguishable.

*Treatment.* The uterus was dilated by means of sponge tents, which, however, gave rise to a mild peritonitis. The depth of the uterine cavity had been increased to one inch. During the period of treatment, there occurred a sanguineous discharge from the vagina. After an interval of five months she resumed treatment, and Dr. Hayes employed the Faradaic current, passing it transversely through the pelvis. Afterwards, the galvanic current from fifteen elements was employed in conjunction with the Faradaic. Improvement was only very slight, and then both currents were applied to the canal and cervix. After twenty-four applications of electricity the uterus appeared to be in the same condition as at the commencement of treatment.

Case II. Amenorrhœa occurring after menstruation had been established, cured by the use of electricity. Mrs. B., colored, twenty-seven years of age, began to menstruate at the age of fifteen and was married at eighteen. Since her marriage her health became much impaired, and her catamenia irregular both as to time and quantity; three or four months frequently intervened between them. She has never been pregnant. She complains of an irritating leucorrhœal discharge, and of pain in the head and in the mammary and ovarian region, and of a frequent and painful desire to micturate; bowels constipated. On examination by Dr. Jackson, it was found that the uterus was in its normal position ; cervix a little elongated ; the os was somewhat nodulated, and had a tenacious mucous plug projecting from it. The sensitiveness of the vaginal walls was abnormal ; the breasts were long and flabby.

*Treatment.* A pill of aloes and myrrh, and electricity. Dr. P. S. Hayes applied the Faradaic current, continued through ten minutes, as follows: one of the electrodes was placed alternately over each ovary and the uterus ; the other electrode over either sacro-iliac synchondrosis, the current being frequently reversed, making the electrodes alternately positive and negative. The treatment was followed by a return of the catamenia, at first scanty and lasting for one day ; the last two each continued three days, and were quite normal in every respect ; the disagreeable subjective symptoms had gradually disappeared. Twelve applications of electricity similar to the one above described, had been given.

Dr. F. H. Davis read a report of two cases of obscure nervous diseases, which had been under the care of Dr. N. S. Davis.

Dr. Bartlett extended an invitation to the members of the Society to examine some fifty slides which he had prepared, containing the blood, secretions, and excretions of ague patients, demonstrating the ague-plant. His investigations have been continued, and results largely increased by recent study in a malarious district of a neighboring State.

